# HCaRG/COMMD5 inhibits ErbB receptor-driven renal cell carcinoma

**DOI:** 10.18632/oncotarget.18012

**Published:** 2017-05-19

**Authors:** Hiroyuki Matsuda, Carole G. Campion, Kyoko Fujiwara, Jin Ikeda, Suzanne Cossette, Thomas Verissimo, Maiko Ogasawara, Louis Gaboury, Kosuke Saito, Kenya Yamaguchi, Satoru Takahashi, Morito Endo, Noboru Fukuda, Masayoshi Soma, Pavel Hamet, Johanne Tremblay

**Affiliations:** ^1^ Centre de recherche, Centre hospitalier de l’Université de Montréal (CRCHUM), Montréal, Québec, Canada, H2X 0A9; ^2^ Department of Medicine, Université de Montréal, Montréal, Québec, Canada, H3T 1J4; ^3^ Division of General Medicine, Department of Internal Medicine, Nihon University, Itabashi-ku, Tokyo, Japan, 173-8610; ^4^ Institut de Recherche en Immunologie et Cancérologie (IRIC), Université de Montréal, Pavillon Marcelle-Coutu, Québec, Canada, H3T 1J4; ^5^ Department of Pathology and Cell Biology, Université de Montréal, Montréal, Québec, Canada, H3T 1J4; ^6^ Department of Urology, Nihon University, Itabashi-ku, Tokyo, Japan, 173-8610; ^7^ Faculty of Human Health Science, Hachinohe Gakuin University, Hachinohe, Aomori, Japan, 031-8588; ^8^ University Research Center, Nihon University, Chiyoda-ku, Tokyo, Japan, 102-8251

**Keywords:** HCaRG/COMMD5, renal cell carcinoma, EGFR, differentiation, autophagic cell death

## Abstract

Hypertension-related, calcium-regulated gene (HCaRG/COMMD5) is highly expressed in renal proximal tubules, where it contributes to the control of cell proliferation and differentiation. HCaRG accelerates tubular repair by facilitating re-differentiation of injured proximal tubular epithelial cells, thus improving mouse survival after acute kidney injury. Sustained hyper-proliferation and de-differentiation are important hallmarks of tumor progression. Here, we demonstrate that cancer cells overexpressing HCaRG maintain a more differentiated phenotype, while several of them undergo autophagic cell death. Its overexpression in mouse renal cell carcinomas led to smaller tumor size with less tumor vascularization in a homograft tumor model. Mechanistically, HCaRG promotes de-phosphorylation of the proto-oncogene erythroblastosis oncogene B (ErbB)2/HER2 and epigenetic gene silencing of epidermal growth factor receptor and ErbB3 via promoter methylation. Extracellular signal-regulated kinase, AKT and mammalian target of rapamycin which mediate ErbB-dowstream signaling pathways are inactivated by HCaRG expression. In addition, HCaRG is underexpressed in human renal cell carcinomas and more expressed in normal tissue adjacent to renal cell carcinomas of patients with favorable prognosis. Taken together, our data suggest a role for HCaRG in the inhibition of tumor progression as a natural inhibitor of the ErbB signals in cancer and as a potential prognostic marker for renal cell carcinomas.

## INTRODUCTION

Hypertension-related, calcium-regulated gene (HCaRG/COMMD5), the longest member of the COMM domain containing (COMMD) protein family, was identified as a gene that is more expressed in the renal proximal tubules (RPT)s of spontaneously-hypertensive than of normotensive rats [1]. Hypertension is not only a well-established risk factor for the progression of renal failure but also increases the risk for renal cell carcinoma (RCC) [2]. Moreover, patients with end-stage renal failure, acquired cystic kidney disease and tubular sclerosis are vulnerable to RCC [3]. Transgenic mice overexpressing HCaRG are more resistant to renal ischemia/reperfusion injury [4]. HCaRG accelerates RPT repair after injury by facilitating re-differentiation and controlling proliferation of injured RPT cells, resulting in a higher mice survival.

HCaRG up-regulation by rosiglitazone was shown to suppress the growth of gastric cancer in the rat model [5]. It is also known that rosiglitazone inhibits RPT cell proliferation through the down-regulation of phosphoinositide 3-kinase (PI3K)/AKT and mitogen-activated protein kinase (MAPK) pathways [6]. The erythroblastosis oncogene B (ErbB) receptors such as epidermal growth factor receptor (EGFR/ErbB1/HER1) and ErbB2, also known as HER2 or p185^c-neu^, are implicated and overexpressed in the development of many types of cancer, and undergo several alterations in human cancers [7, 8]. Inhibitors targeting the ErbB2 selective tyrosine kinase and combined blockade of PI3K/AKT and MAPK pathways inhibit tumor growth including RCC [9, 10]. A recent study reported that HCaRG and other COMMD proteins were decreased in non-small cell lung cancer, whereas increased COMMD9 promoted cell proliferation, migration and cell-cycle progression, and inhibited autophagy via transcription factor Dp-1/E2F transcription factor 1 activation [11]. Furthermore, COMMD1 has been shown to be suppressed in human cancer and its decreased expression to correlate with a more invasive tumor phenotype [12].

We hypothesized that a low level of HCaRG expression contributes to uncontrolled cell proliferation and de-differentiation in RCC. We demonstrate here that HCaRG is less expressed in RCC and that its overexpression prevents tumorigenesis and angiogenesis of RCC in a homograft mouse model and enhances autophagic cell death. Finally, we report here that the inhibition of tumor development by HCaRG is mediated by inactivation of ErbB signalling.

## RESULTS

### HCaRG inhibits cell-cycle progression and facilitates differentiation

**Figure 1:**
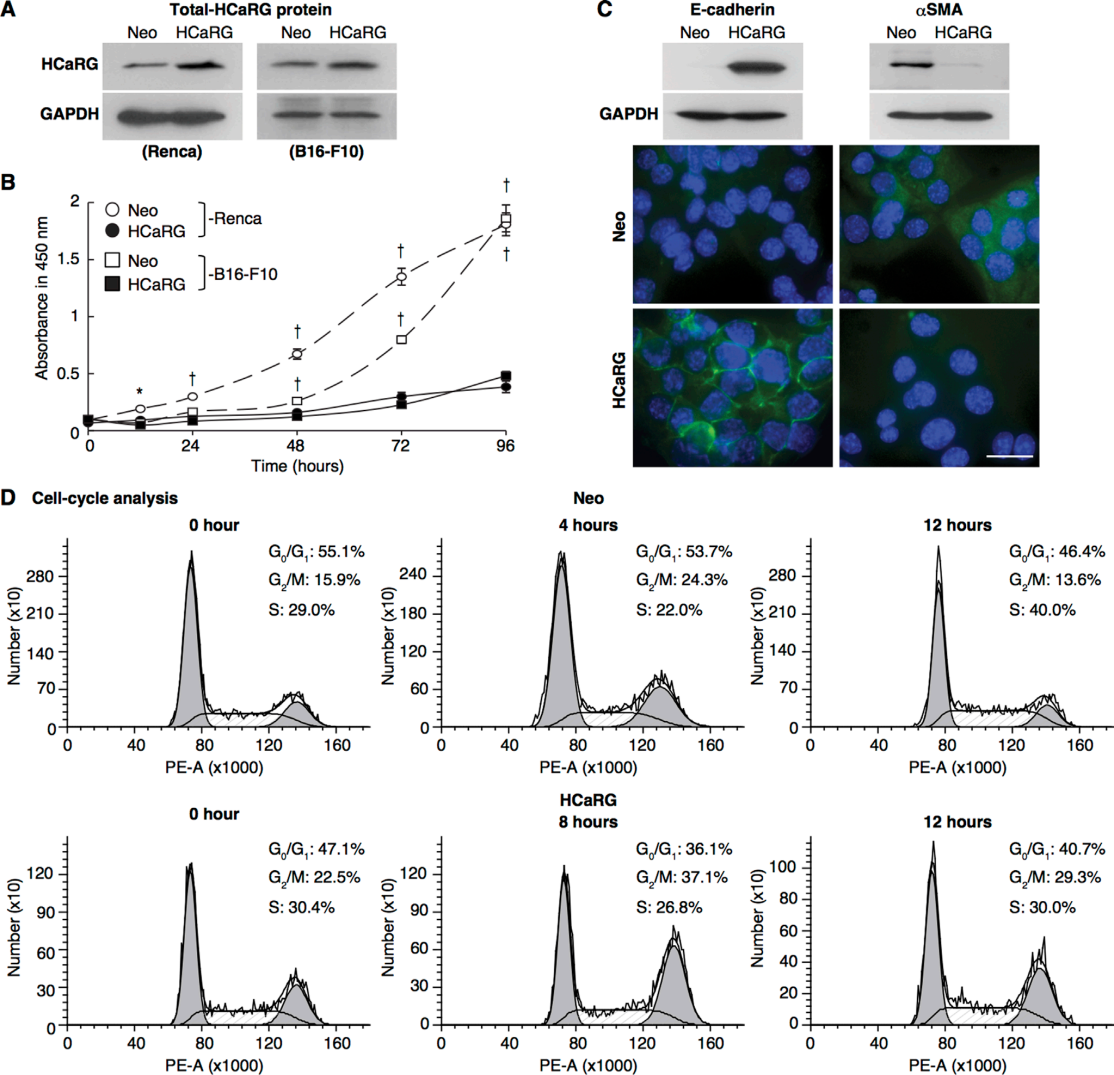
HCaRG facilitates differentiation of Renca cells and inhibits cell-cycle progression (**A**) Total (exogenous and endogenous) HCaRG protein levels, revealed by western blot, were higher in HCaRG-Renca and HCaRG-B16-F10 cells than in Neo-control cells. (**B**) Cell growth curves of Renca and B16-F10 clones. HCaRG-Renca and HCaRG-B16-F10 cells proliferated less than Neo-controls. ^†^*P* < 0.005. (**C**) Western blot and immunostaining of differentiation markers in Renca clones. E-cadherin was detected only in HCaRG-Renca cells while αSMA expression was lower in HCaRG-Renca than in Neo-Renca cells. HCaRG promoted differentiation of Renca cells. Scale bars, 50 µm. (**D**) Cell-cycle analysis by DNA content in Renca cells. Representative cell-cycle DNA histograms showed the effect of HCaRG overexpression on cell-cycle progression in Renca cells. The maximal peak of G_2_/M accumulation was observed at 4 hours in Neo-Renca and at 8 hours in HCaRG-Renca cells, respectively. HCaRG delayed cell-cycle progression by 4 hours with G_2_/M cell-cycle accumulation. G_0_/G_1_, G_2_/M and S phases are shown.

To test whether HCaRG might act as a tumor-suppressor gene, we initially evaluated the impact of its overexpression in a mouse renal adenocarcinoma cell line, Renca cells and a melanoma cell line, B16-F10 cells. Cells were stably transfected with control plasmid (Neo-Renca and Neo-B16-F10) or an expression plasmid containing rat HCaRG cDNA (HCaRG-Renca and HCaRG-B16-F10) as described previously [13]. As expected, rat HCaRG mRNA could be detected only in HCaRG-Renca and HCaRG-B16-F10 cells (Supplementary Figure 1A), and HCaRG protein levels were higher in stably transfected HCaRG-Renca and HCaRG-B16-F10 cells than in Neo-control cells (Figure 1A). HCaRG overexpression inhibited cell proliferation of both Renca and B16-F10 cells as we previously reported for other cell types (Figure 1B) [1]. While HCaRG produced similar effects in two different cancer cell lines, the current study focused on RCC. Neo-Renca cells were spindle shaped and expressed high levels of α-smooth muscle actin (αSMA), a marker of mesenchymal phenotype [14], whereas HCaRG-Renca cells had a more cobblestone-like epithelial shape and expressed E-cadherin, a marker of epithelial integrity (Figure 1C) [15].

Cell-cycle analysis of synchronized Neo- and HCaRG-Renca cells is shown in Figure 1D and Table 1. The cell-cycle length was approximately 8 hours in Neo-Renca cells. HCaRG overexpression increased cell cycle length by more than 4 hours. These data demonstrate that HCaRG inhibited cell proliferation by augmenting cell-cycle length with G_2_/M cell-cycle accumulation and facilitating differentiation of Renca cells. Similar results were obtained with B16-F10 cells (Supplementary Figure 1B).

**Table 1:** Cell-cycle analysis by DNA content in Renca cells

Time (hour)		0	4	8	12	24
**Neo**	G_0_/G_1_ (%)	55.1	53.7	60.1	46.4	52.0
	G_2_/M (%)	15.9	24.3	16.3	13.6	15.6
	S (%)	29.0	22.0	23.6	40.0	32.4
**HCaRG**	G_0_/G_1_ (%)	47.1	37.4	36.1	40.7	39.6
	G_2_/M (%)	22.5	23.5	37.1	29.3	26.8
	S (%)	30.4	39.1	26.8	30.0	33.6

### HCaRG enhances autophagic cell death

**Figure 2:**
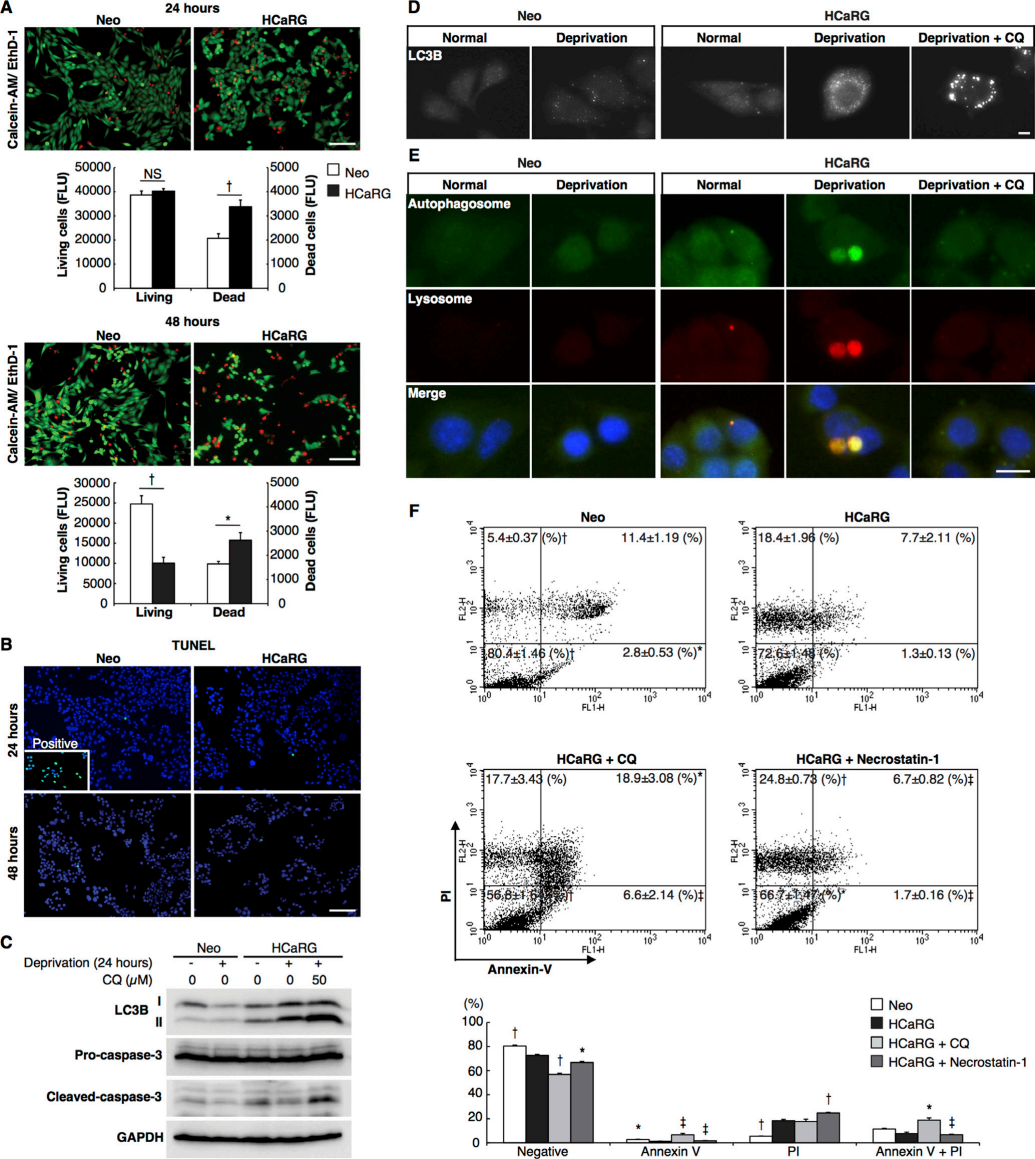
HCaRG induces autophagic cell death distinct from apoptosis or necroptosis in Renca cells (**A**) Living cells and nuclei of dead cells were double-stained with calcein-AM (green) and EthD-1 (red), respectively, for relative fluorescence (FLU) quantification. HCaRG overexpression increased the number of dead cells compared to Neo-controls. Serum deprivation decreased the number of surviving cells in HCaRG-Renca cells compared to Neo-controls after 48 hours. ^†^*P* < 0.005. Scale bars, 100 µm. NS, not significant. (**B**) TUNEL staining in synchronized Renca cells grown in serum depleted medium. Only a few apoptotic cells could be detected with no differences between Neo- and HCaRG-Renca cells relative to the DNase I-treated positive control Renca cells. Scale bars, 100 µm. (**C**) Western blots of LC3B and caspase-3 protein levels in Renca cells. The expression of LC3B-II was increased by HCaRG overexpression 24 hours after serum deprivation. Pro-caspase-3 levels were not different between Neo- and HCaRG-Renca cells. Cleaved-caspase-3 was reduced by serum deprivation in both Neo- and HCaRG-Renca cells. Inhibition of autophagy by CQ increased LC3B-II and cleaved-caspase-3 expression. (**D**) Immunofluorescence staining of LC3B in Renca cells. LC3B puncta were higher in HCaRG-Renca than Neo-cells after 3 hours serum deprivation. CQ treatment increased the number of enlarged LC3B puncta in HCaRG-Renca cells. Scale bars, 10 µm. (**E**) Fluorescent stains of autophagosomes and lysosomes in Renca cells. Large autolysosomes (autophagosome-lysosome fusion) were detected only in HCaRG-Renca after 3 hours of serum deprivation. CQ treatment inhibited the formation of autolysosomes in HCaRG-Renca cells. Scale bars, 10 µm. (**F**) Annexin-V/PI staining was performed to quantify the dead cell population. There were more PI-positive and Annexin V-negative necrotic cells in HCaRG-Renca cells than in Neo-Renca cells. HCaRG overexpression decreased dual positive apoptotic cells, in parallel. CQ treatment decreased significantly more living cells with a higher percentage of apoptotic cells than serum deprivation in HCaRG-Renca cells. Inhibition of necroptosis by Necrostatin-1 treatment increased the number of necrotic cells. The percentage of cells in each quadrant is indicated as mean ± SD. ^‡^*P* < 0.05, *P < 0.01, ^†^*P* < 0.005 compared to starved HCaRG-Renca controls.

To assess the impact of HCaRG on cell death, both Neo- and HCaRG-Renca cells were seeded so as to reach the same number of living cells 24 hours after serum deprivation and were double-stained with calcein-AM and ethidium homodimer (EthD)-1 for relative fluorescence quantification (Figure 2A). The number of dead cells was 1.6-fold higher in HCaRG-Renca than in Neo-Renca cells. After 48 hours deprivation, the number of surviving HCaRG-Renca cells was 40% of Neo-Renca cells. HCaRG produced the same effect in B16-F10 cells (Supplementary Figure 2A). Programmed cell death (PCD), a regulated process crucial to organ homeostasis and tumor suppression, can be classified into several types that include apoptosis, cell death with autophagy and necroptosis [16]. In comparison with DNase I-treated positive control cells, there were only a few apoptotic cells with no differences between Neo- and HCaRG-Renca cells by the terminal deoxynucleotidyl transferase–mediated dUTP nick-end labeling (TUNEL) assay 24 and 48 hours after serum deprivation (Figure 2B), and none in B16-F10 cells (Supplementary Figure 2B). The activation of the proapoptotic mediator, caspase-3 was inhibited to the same extent 24 hours after serum deprivation (Figure 2C), suggesting that HCaRG had no impact on Renca cell apoptosis.

We then tested whether HCaRG induced autophagy. The expression of microtubule-associated protein 1 light chain 3 (LC3B)-II as well as LC3B puncta, a widely used marker of autophagosome [17], were markedly increased by serum deprivation in HCaRG-Renca and HCaRG-B16-F10 but not in Neo-control cells (Figure 2C and 2D, Supplementary Figure 2C and 2D). The inhibition of autophagy by treatment with chloroquinone (CQ), which is a pharmacological agent capable of impairing lysosomal acidification [18], caused a further increase in the amount of LC3B-II protein relative to the LC3B-I and enlarged LC3B puncta in HCaRG-Renca cells. As expected, cleaved-caspase-3 level was increased in HCaRG-Renca cells treated with CQ (Figure 2C). Moreover, large autolysosomes that were formed by the fusion of autophagosomes stained with Cyto-ID Green and lysosomes detected by LysoTracker Red probe were observed only in HCaRG-Renca cells, while the punctate distribution of autophagosomes did not co-localize with lysosomes in HCaRG-Renca cells treated with CQ treatment (Figure 2E). Flow cytometry was used to analyze dead cell sub-populations. As shown in Figure 2F, HCaRG overexpression significantly decreased dual-negative living cells. HCaRG-Renca cells showed a higher percentage of propidium iodide (PI)-positive/Annexin-V-negative necrotic cells with a reduced level of early (PI-negative/Annexin-V-positive) and late (dual-positive) apoptotic cells than Neo-Renca cells. Treatment of HCaRG-Renca cells with CQ increased mortality with a higher percentage of apoptotic cells than serum deprivation alone. These data indicate that the high level of autophagy induced by HCaRG overexpression under serum deprivation is associated with reduced rather than enhanced cell death.

To exclude the possibility that HCaRG-induced cell death is due to necroptosis, we incubated HCaRG-Renca cells with Necrostatin-1, which suppresses necroptosis by inhibiting receptor-interacting protein-1 kinase activity [19, 20]. Necrostatin-1 treatment decreased the number of living HCaRG-Renca cells with a parallel increase in the number of necrotic cells (Figure 2F). We can therefore conclude that HCaRG expression in Renca cells does not promote apoptosis or necroptosis but rather fosters autophagic cell death.

### HCaRG inhibits tumor growth and angiogenesis

**Figure 3:**
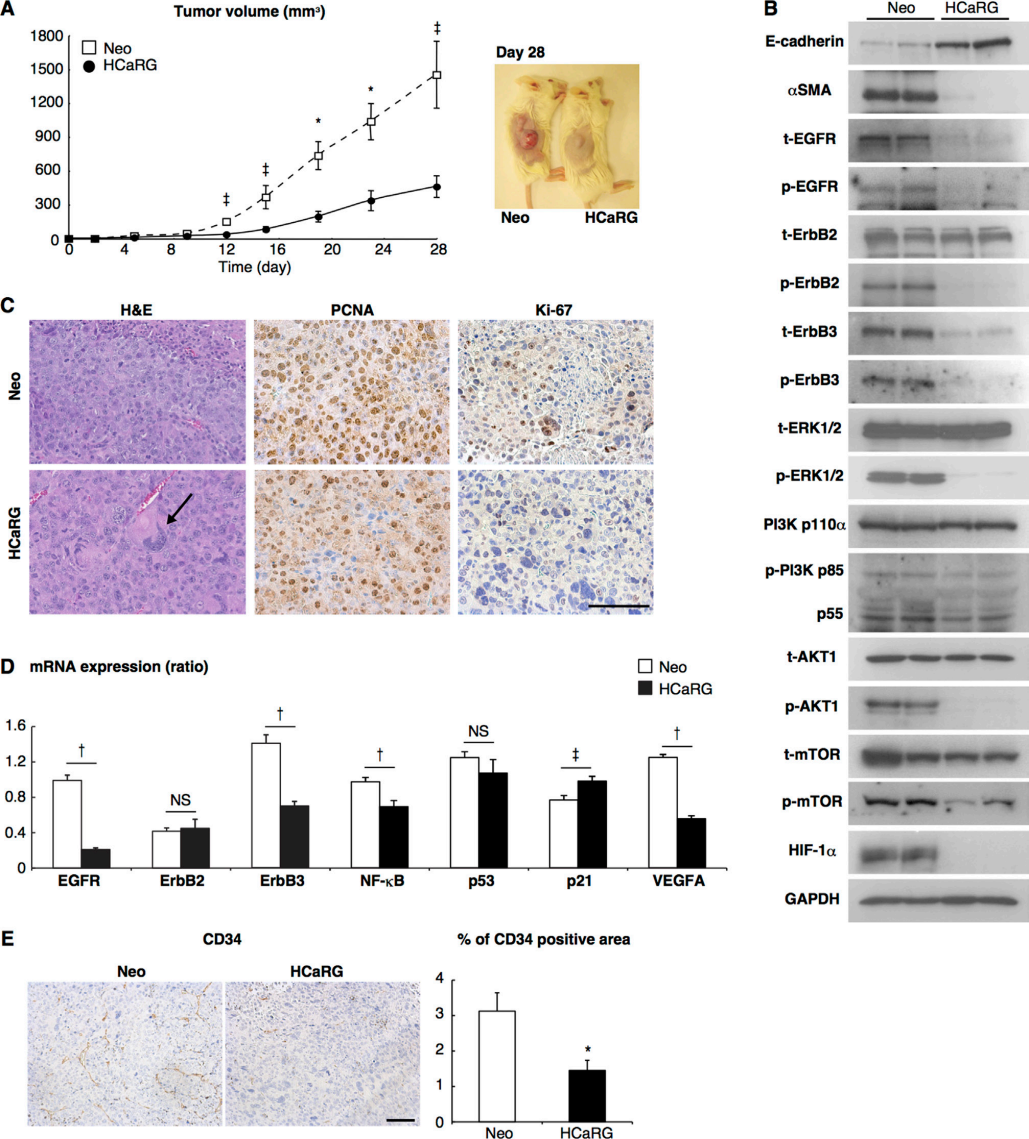
HCaRG inhibits tumor growth in a mouse homograft RCC model (**A**) Renca clones were implanted by subcutaneous injection (1 × 10^6^ cells/mouse). HCaRG overexpression significantly inhibited tumor growth of homografted Renca cells as seen after 8 days. ^‡^*P* < 0.05, *P < 0.01. (**B**) Tumor lysates obtained 28 days after implantation were analyzed by western blot using appropriate antibodies. HCaRG overexpression led to more differentiated tumor cells in experimental RCCs, as indicated by more E-cadherin and less of αSMA compared to Neo-controls. Representative blots showed further that HCaRG suppresses EGFR and ErbB3. ErbB2 protein level was not changed by HCaRG overexpression, while its phosphorylation was diminished. The subsequent MAPK and PI3K/AKT pathways were inactivated in HCaRG-RCCs. (**C**) Representative images of H&E stain and immunostaining with two different proliferation markers, PCNA and Ki-67. HCaRG-RCCs showed less cell proliferation with increased multinucleated giant cells relative to Neo-RCCs. The black arrow indicates a multinucleated giant cell. Scale bars, 100 µm. (**D**) The mRNA expressions of ErbB receptors and their downstream genes were demonstrated by Real-Time PCR. ^‡^*P* < 0.05, ^†^*P* < 0.005. NS, not significant. (**E**) Representative images and quantitative data of immunostaining with anti-CD34-antibody. HCaRG overexpression markedly decreased CD34-positive microvessels and endothelial cells in experimental RCCs at day 28. *P <0.01. Scale bars, 100 µm.

We then evaluated the effects of HCaRG on tumor growth *in vivo* using a homograft tumor model. Six syngeneic BALB/c mice per group were injected subcutaneously with HCaRG-Renca cells (HCaRG-RCC) or Neo-Renca cells (Neo-RCC). The size of the tumors was measured 28 days after injection. The tumor size of HCaRG-RCCs was 32% of the tumor size of Neo-RCCs (Figure 3A). HCaRG-RCCs also appeared to be more differentiated than Neo-RCC as demonstrated by their higher content in E-cadherin and lower αSMA levels (Figure 3B). In addition, hematoxylin and eosin (H&E) staining of tumors showed a higher number of multinucleated giant cells in HCaRG-RCCs compared to Neo-RCCs (Figure 3C). We corroborated that HCaRG overexpression inhibited cell proliferation in RCCs by immunostaining with the proliferating nuclear cell antigen (PCNA) and Ki-67 (Figure 3C). These data suggest that HCaRG inhibited tumor growth of RCCs by maintaining a more differentiated phenotype and by controlling cell proliferation, suggesting a role of HCaRG as a tumor-suppressor gene in RCC.

We concurrently examined the effects of HCaRG on angiogenesis in experimental RCCs. T*umor* angiogenesis is induced by the *secretion* of various g*rowth factors including* platelet-derived growth factor (PDGF) and vascular endothelial growth factor (VEGF). PI3K/AKT signaling pathway controls these angiogenic factors through the activation of transcriptional regulator hypoxia inducible factor (HIF)-1α [21, 22]. We showed that HIF-1α expression was diminished in HCaRG-RCCs (Figure 3B). We then demonstrated subsequent reduction of VEGFA mRNA levels in HCaRG-RCCs (Figure 3D) and serum VEGF concentration in HCaRG-RCC mice (Table 2). To confirm the anti-angiogenic effect of HCaRG in experimental RCCs, we performed immunohistochemical staining of tumor microvessels using anti-CD34-antibody (Figure 3E) [22]. HCaRG-RCCs clearly had less CD34-positive microvessels and endothelial cells relative to Neo-RCCs. These data show that HCaRG overexpression negatively controlled the transcription of VEGF by down-regulating HIF-1α, thus reducing tumor angiogenesis.

**Table 2:** Serum VEGF concentration at day 28 (pg/ ml)

Sham	Neo	HCaRG
112.3 ± 10.3 (*n =* 5)	135.1 ± 8.4 (*n =* 6)*	116.5 ± 17.2 (*n =* 6)^‡^

Data are shown as mean ± SD. *P < 0.01 vs Sham control mice. ^‡^*P* < 0.05 vs mice with Neo-RCCs.

### HCaRG targets ErbB receptors and their signaling pathways

Oncogene amplification and overexpression of ErbB receptors have been observed in many human tumors of epithelial origin, and have been linked to cancer development and progression [23]. We thus examined the effect of HCaRG on the expression/activation of ErbB receptors and their downstream signaling pathways. HCaRG overexpression dramatically inhibited the expression/phosphorylation of EGFR and ErbB3 in experimental RCCs (Figure 3B). Interestingly, while ErbB2 expression was not different between Neo- and HCaRG-RCCs, its phosphorylation was abolished by HCaRG overexpression.

**Figure 4:**
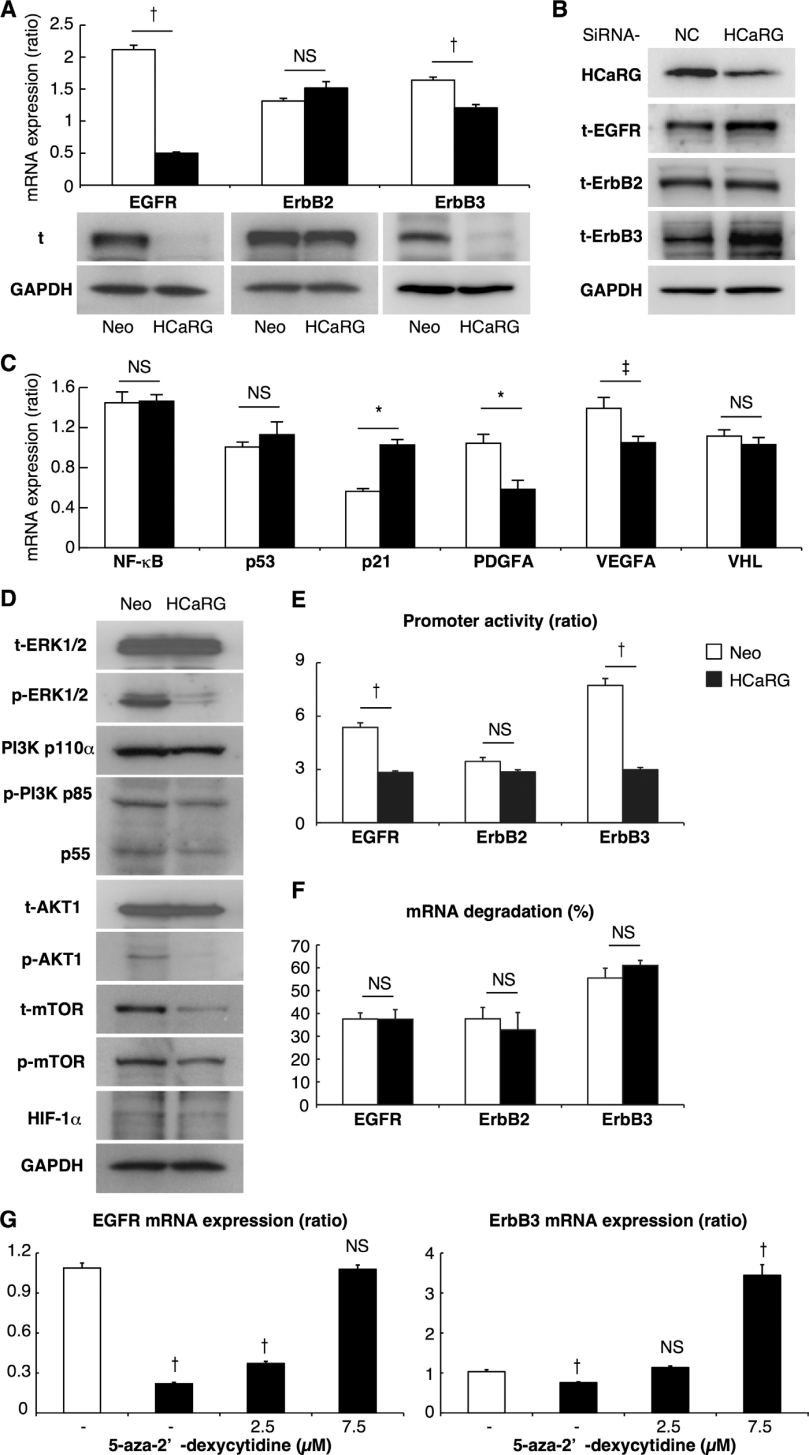
HCaRG suppresses EGFR and ErbB3 by enhancing their promoter methylation (**A**) The expression of total (t) ErbB receptors was demonstrated by Real-Time PCR and western blot. HCaRG overexpression reduced EGFR and ErbB3 expression at the mRNA and protein levels in Renca cells. ErbB2 mRNA and total protein levels were not modified by HCaRG overexpression. ^†^*P* < 0.005. NS, not significant. (**B**) Knockdown of HCaRG with siRNA treatment in WT-Renca cells. siRNA-HCaRG treatment reduced HCaRG protein expression compared to a negative control (NC) and led to up-regulation of EGFR and ErbB3. ErbB2 expression was not changed by siRNA against HCaRG. (**C**) The effect of inhibition of MAPK and PI3K/AKT signals on downstream genes was demonstrated by Real-Time PCR in Renca cells. The decrement of HIF-1α and subsequent VEGFA and PDGF genes was caused by HCaRG overexpression independent of the expression of anti-oncogene of RCC, VHL. ^‡^*P* < 0.05, *P < 0.01, ^†^*P* < 0.005. NS, not significant. (**D**) Renca lysates were analyzed by western blot using appropriated antibodies. Representative blots show the inactivation of subsequent MAPK and/or PI3K/AKT signaling pathways by HCaRG overexpression. (**E**) HCaRG inhibits the transcriptional activity of EGFR and ErbB3 at the promoter level. Human EGFR, ErbB2 and ErbB3 5′ flanking regions containing their respective promoters were cloned and transfected into Neo- or HCaRG-Renca cells. HCaRG overexpression repressed the promoter activities of EGFR and ErbB3, but not of ErbB2. Promoter activity was normalized to the activity of pGL4.74-TK control vector. ^‡^*P* < 0.05, ^†^*P* < 0.005. (**F**) mRNA degradation of ErbB receptors was tested by inhibiting their transcription with α-amanitin. mRNA levels were normalized to polymerase I-dependent transcription of the 28S ribosomal gene. mRNA degradation was presented as percentage of mRNA levels relative to controls without α-amanitin. HCaRG did not cause mRNA degradation after 24 hours. (**G**) Demethylation of promoter DNA with treatment of 5-aza-2’-deoxycytidine rescued and/or increased mRNA levels of EGFR and ErbB3 in HCaRG-Renca cells in a dose-dependent manner. ^‡^*P* < 0.05, ^†^*P* < 0.005.

The MAPK and PI3K/AKT represent the major ErbB signaling pathways [7, 23]. HCaRG overexpression inhibited these signaling pathways in experimental RCCs as illustrated in Figure 3B. More precisely, HCaRG overexpression did not reduce extracellular signal-regulated kinase (ERK)1/2 expression, but inhibited its phosphorylation. The most important regulators of PI3K are the catalytic subunit p110α and its associated regulatory subunit p85/p55 [24]. Again, HCaRG overexpression did not reduce p110α expression, but inhibited p85/p55 phosphorylation. Similarly, AKT level was not decreased by HCaRG overexpression but its phosphorylated state was diminished. The levels of p53, which acts as a p21 transcriptional activator [25], were not different between Neo- and HCaRG-RCCs, while p21 was increased in HCaRG-RCCs independently of p53 (Figure 3D). In addition, the mRNA levels of nuclear factor (NF)-κB, which is indirectly controlled by AKT and stimulates cell survival, proliferation and angiogenesis in cancers [24, 26], was significantly lower in HCaRG-RCCs relative to Neo-RCCs. These data suggest that the MAPK and PI3K/AKT signaling pathways were inactivated as a consequence of the down-regulation of EGFR and ErbB3 by HCaRG overexpression. The inhibitory effect of HCaRG on ErbB expression was also seen in cultured Renca cells (Figure 4A). Similar pattern of ErbB receptor expression was seen in B16-F10 cells, with the exception of EGFR that was not detectable in this cell line (Supplementary Figure 3A). Furthermore, we showed that this effect is specific to HCaRG expression as knockdown of HCaRG by small interfering RNA (siRNA) increased EGFR and ErbB3 expressions in wild-type (WT)-Renca cells (Figure 4B), suggesting that HCaRG controls the expression of EGFR/ErbB receptor family and their subsequent signaling pathways in Renca and B16-F10 cells (Figure 4C and 4D, Supplementary Figure 3B and 3C).

### HCaRG leads to DNA hyper-methylation of EGFR and ErbB3

**Figure 5:**
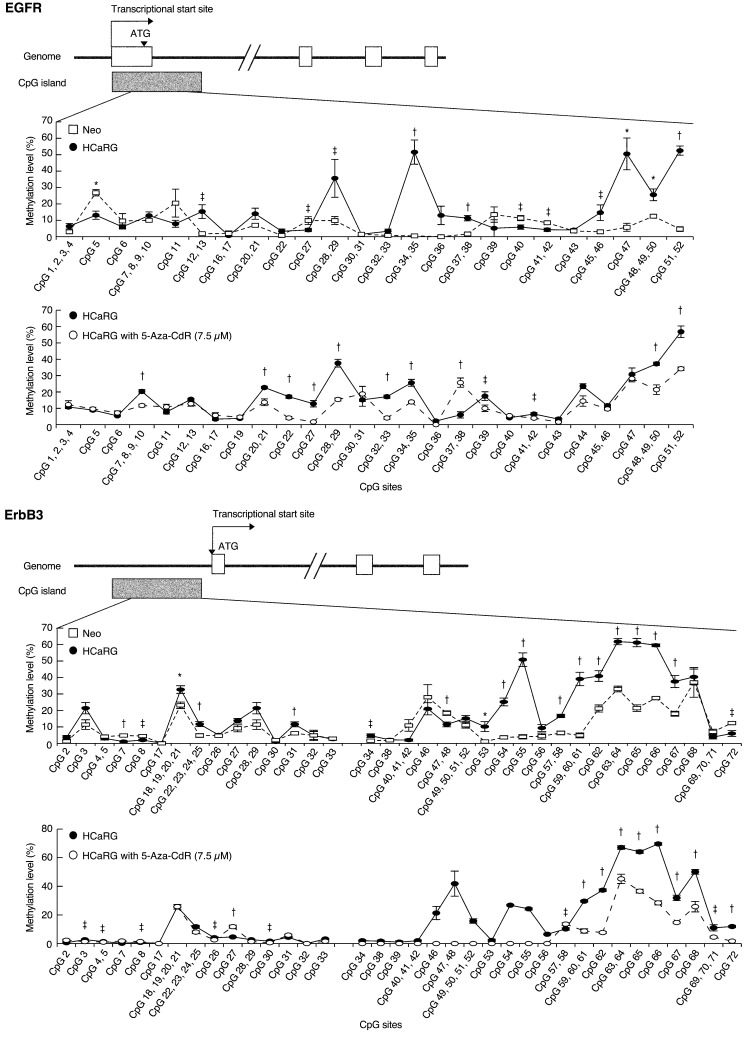
HCaRG fosters CpG island hyper-methylation of EGFR and ErbB3 promoters Methylation level of CpG island located in EGFR and ErbB3 promoters was quantified using MassARRAY quantitative methylation analysis in Renca clones. Both EGFR and ErbB3 promoters showed significantly higher methylation levels in HCaRG-Renca cells than in Neo-Renca cells. Several hyper-methylated CpG sites located in EGFR and ErbB3 promoters in HCaRG-Renca cells were de-methylated 72 hours after treatment of 5-Aza-CdR. ^‡^*P* < 0.05, *P < 0.01, ^†^*P* < 0.005.

To investigate the mechanism by which HCaRG suppresses ErbB receptors, we performed luciferase reporter assays for human EGFR, ErbB2 and ErbB3 promoters (Figure 4E). Promoter activities of EGFR and ErbB3 but not of ErbB2 were significantly lower in HCaRG-Renca cells than in Neo-controls while their mRNA degradation was not different (Figure 4F), suggesting that HCaRG effects occur at the transcriptional level. We then observed that the diminished EGFR and ErbB3 mRNA levels in HCaRG-Renca cells were rescued and/or increased by treatment with 5-aza-2’-deoxycytidine (5-Aza-CdR), which is a demethylating agent that inhibits DNA methyltransferase activity, in a dose-dependent manner (Figure 4G). In fact, several CpG sites located in the EGFR and ErbB3 promoters showed higher methylation levels in HCaRG-Renca cells than in Neo-Renca cells (Figure 5), and these hyper-methylated CpG sites in HCaRG-Renca cells could be demethylated by 5-Aza-CdR treatment. These data indicate that a hyper-methylated state in EGFR and ErbB3 promoters could result in their transcriptional gene silencing observed in HCaRG-Renca cells.

### HCaRG levels in RPTs predict survival of patients with RCC

**Figure 6:**
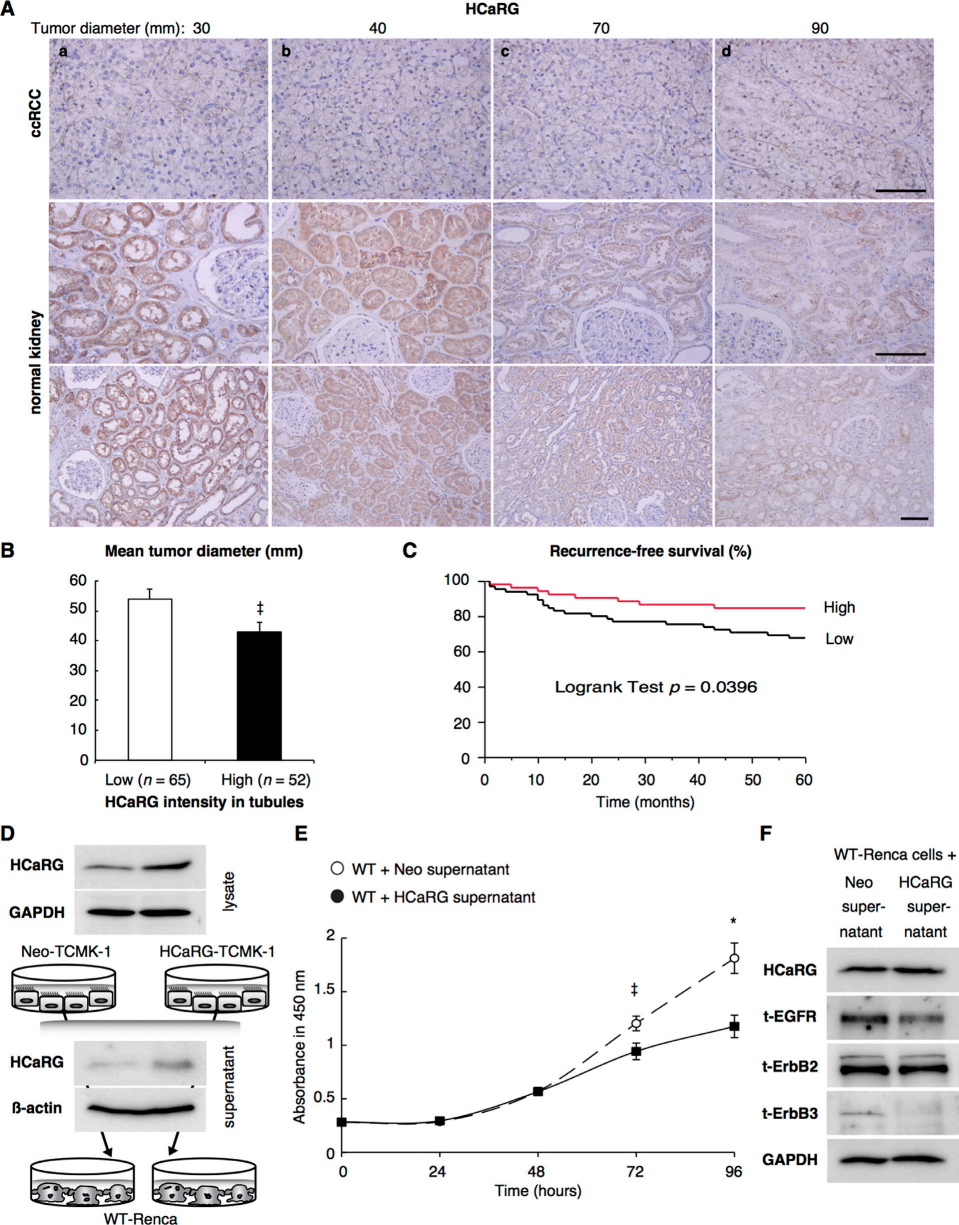
HCaRG is less expressed in human ccRCCs than in normal renal tubules (**A**) Representative HCaRG immunohistochemical staining was performed on sections that included not only ccRCC (upper panels) but also normal kidney tissues adjacent to tumors. Strong HCaRG intensity was recorded in normal kidneys adjacent to smaller ccRCCs (maximum tumor diameter 30 and 40 mm) as in a and b. HCaRG staining intensity was weak in ccRCCs and renal tubules from patients with larger tumor size (maximum tumor diameter 70 and 90 mm) as in c and d. Scale bars, 100 µm. (**B**) High HCaRG levels in normal renal tubules were associated with small tumor size of ccRCCs (^‡^*P* < 0.05). Patients were classified into high and low HCaRG levels in renal tissues adjacent to tumors. (**C**) 5-year recurrence-free survival curves of ccRCC patients. High HCaRG levels in normal renal tubules are a predictor of better prognosis. (**D**) Total (exogenous and endogenous) HCaRG protein levels in cell lysates and acetone-precipitated supernatants of culture media from mouse kidney epithelial cells, TCMK-1 clones, revealed by western blot, were higher in HCaRG-TCMK-1 cells than in Neo-controls. HCaRG protein was secreted by renal tubular epithelial cells. (**E**) Cell growth curve of WT-Renca cells incubated with cell-culture supernatant from Neo-TCMK-1 or HCaRG-TCMK-1 cells. Cell proliferation of WT-Renca cells incubated with cell-culture supernatant of HCaRG-TCMK-1 cells was inhibited compared to cells incubated with cell-culture supernatant of Neo-TCMK-1 cells. ^‡^*P* < 0.05, *P < 0.01. (**F**) WT-Renca cells were incubated in cell-culture supernatant of Neo- or HCaRG-TCMK-1 cells for 96 hours. The cell-culture supernatant from HCaRG-TCMK-1 cells reduced the protein levels of EGFR and ErbB3 compared to cells incubated in cell-culture supernatant of Neo-TCMK-1 cells. Endogenous HCaRG and ErbB2 expression levels were not changed by these cell-culture supernatants.

Human clear-cell RCCs (ccRCCs) and normal tissues adjacent to tumors were immunohistochemically stained with anti-HCaRG-antibody (Figure 6A). HCaRG levels were much lower in ccRCCs tumors than in their paired normal tissues. The strongest HCaRG staining intensity was seen in normal RPTs around tumors of smaller size. The compilation of ccRCCs from 117 subjects is shown in Figure 6B and Table 3. Classification of normal RPTs into high (strong staining) and low (weak/absence staining) levels of HCaRG showed a significant difference in tumor diameter (*P* = 0.0362), pathological T stage (*P* = 0.0041), Fuhrman grade (*P* = 0.0085) and recurrence within 5 years (*P* = 0.0296). HCaRG levels in normal RPTs were negatively correlated with tumor size and nuclear grade. The correlation of HCaRG expression in normal tissues with survival was also analyzed. Figure 6C illustrates the recurrence-free survival curves during 5 years of patients classified into low and high HCaRG groups. Low HCaRG levels in normal tissues were significantly (*P* = 0.0396) associated with worse clinical outcome.

**Table 3:** Clinicopathological features of the 117 ccRCCs patients and their correlations with HCaRG expression in normal RPTs

Parameter		HCaRG expression		*P* value
		High (*n =* 52)	Low (*n =* 65)	
**Maximum diameter (mm)**		42.9 ± 23.3 SD	54.0 ± 26.9 SD	0.0205
**Age (year)**		61.8 ± 10.3 SD	59.7 ± 11.4 SD	0.291
**Gender**	Male	42	41	0.0362
	Female	10	24	
**Pathological T stage**	T1a (≤ 4 cm)	33	24	0.0041
	≥ T1b (> 4 cm)	19	41	
**Pathological N stage**	N0	52	64	1
	N+	NA	1	
**M stage**	M0	48	56	0.2926
	M+	4	9	
**Fuhrman Grade**	1	24	15	0.0085
	≥ 2	28	50	
**Recurrence within 5 years**	+	7	20	0.0296
	free	45	45	

NA: not applicable.

Since high expression level of HCaRG in RPTs could be involved in the slow growth rate of ccRCC, we lastly determined if HCaRG secreted by RPTs inhibits tumor growth. For this purpose, we transfected mouse kidney epithelial cells with control plasmid (Neo-TCMK-1) or rat-HCaRG expression plasmid (HCaRG-TCMK-1). As expected, both intracellular and secreted HCaRG protein levels were higher in HCaRG-TCMK-1 cells than in Neo-TCMK-1 cells (Figure 6D). Cell-culture supernatants of HCaRG-TCMK-1 cells inhibited cell proliferation of WT-Renca cells after 48 hours compared to cells incubated with cell-culture supernatant of Neo-TCMK-1 cells (Figure 6E). The expression of EGFR and ErbB3 was also suppressed in WT-Renca cells incubated with cell-culture supernatant of HCaRG-TCMK-1 cells after 96 hours, while endogenous HCaRG and ErbB2 expression was not different (Figure 6F).

## DISCUSSION

About 270,000 people worldwide are diagnosed with kidney cancer every year and nearly 120,000 die from it annually. Until the underlying pathophysiological mechanisms of renal oncogenesis is known, partial or radical nephrectomy will remain the main curative treatment for localized RCC while the current therapy for advanced RCC is still inadequate [3, 27–29]. We report here that HCaRG reduced tumor enlargement and facilitated differentiation in RCC through the inactivation of the ErbB receptor tyrosine kinase family. Upon binding their specific ligands, ErbB receptors undergo homodimerization and/or heterodimerization, and subsequent transphosphorylation of their intrinsic kinase domain. Despite the fact that there is no known extracellular ligand for ErbB2, it is the preferred heterodimerization partner of all the other ErbB receptors [7, 30]. Due to its enhanced expression in cancers and involvement in essential signaling processes, ErbB2 constitutes an important target for cancer therapy. We found that HCaRG overexpression markedly inhibited ErbB2 phosphorylation without reducing its protein level. The other ErbB receptors, EGFR and ErbB3, were suppressed by HCaRG overexpression at the transcriptional levels through the modification of the methylated status of their promoter regions, and conversely knockdown of HCaRG increased EGFR and ErbB3 expression. These data suggest that the increased methylation status of EGFR and ErbB3 promoters induced by HCaRG overexpression results in a reduction of their protein levels and subsequent reduced capacity to heterodimerize with ErbB2. Widespread DNA hyper- or hypo-methylation are associated with underlying gene mutations of the PI3K/AKT/mTOR signaling pathway in ccRCC [31].

**Figure 7:**
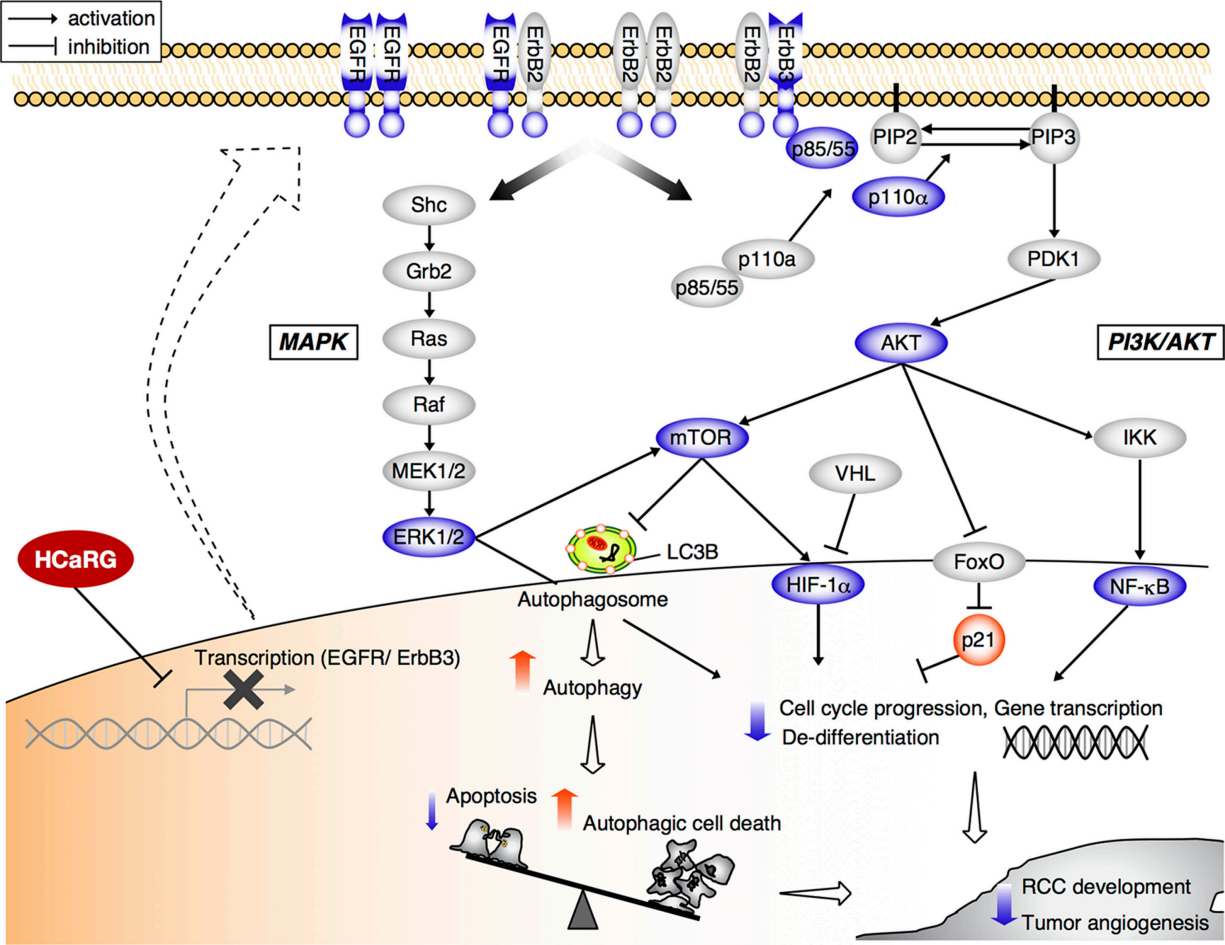
Scheme depicting the suppressive role of HCaRG in tumorigenic pathways of RCC Cancer cells overexpressing HCaRG show a more differentiated phenotype with lower cell proliferation than Neo-controls. HCaRG inhibits the phosphorylation of the proto-oncogene ErbB2 and inactivates subsequent MAPK and PI3K/AKT pathways by inducing the epigenetic gene silencing of EGFR and ErbB3 genes through their promoter methylation (Me). In addition, HCaRG facilitates programmed cell death by inducing autophagy via inactivation of AKT/mTOR pathway. As a result, tumor growth and angiogenesis of RCC are inhibited by HCaRG. The blue color indicates down-regulation or de-phosphorylation. The red color indicates up-regulation or activation.

Recent advances into the molecular understanding of RCC have contributed to the development of novel molecularly targeted therapeutics such as VEGF-antibody and small molecules interfering with tyrosine kinase receptors and mammalian target of rapamycin (mTOR) [32, 33]. Our studies also demonstrate that HCaRG overexpression inactivates the MAPK and PI3K/AKT signaling pathways downstream of ErbB receptors in renal carcinoma and melanoma cells (Figures 3 and 4, Supplementary Figure 3). In cancer, these crucial pathways are frequently inhibited, resulting in perturbations in downstream targets that deregulate growth factors and cell survival signals [24, 30, 34]. We thus conclude that HCaRG has a potential for tumor suppression not only in ccRCC but also in other ErbB- or PI3K/AKT signaling-driven cancers including melanoma. In our previous reports, HCaRG inhibited cell proliferation and facilitated differentiation through the induction of p21, transactivated by a p53-independent pathway [4, 35]. p21 induction has be shown to be controlled by the PI3K/AKT pathway through nuclear export of nuclear forkhead box (FoxO) proteins [24]. Furthermore, the AKT/mTOR signals lead to increased expression of HIF-1α and subsequent angiogenic factors including VEGF and PDGF [30, 36]. Under normal conditions, HIF-1α is degraded by von Hippel-Lindau gene (VHL), however its levels are abnormally high in ccRCC due to dysfunction or absence of VHL [37]. In our study, HCaRG overexpression significantly reduced tumor vascularization most probably by suppressing VEGF through the inactivation of AKT/mTOR/HIF signaling independently of abnormal VHL expression (Figure 4C). Our results support the notion that lower HCaRG expression observed in ccRCC may contribute to tumor-associated sustained proliferative phenotypes and neovasculature, thus resulting in rapid tumor progression (Figure 7).

Evading PCD is one of the critical features of tumorigenesis. PCD can be caused not only by apoptotic signals but also by necroptosis and autophagic cell death which is defined by morphological characteristics such as autophagosome/autolysosome formation [16, 38, 39]. In this study, HCaRG induced autophagy under serum deprivation most probably through the inactivation of PI3K/AKT/mTOR pathway. Autophagy can help cancer cells to survive under hypoxia or insufficient nutritional condition [40]. Over the past decade, many studies have shown that autophagy is critically important for the survival, activation and differentiation of multiple cell types. During malignant transformation, as well as in response to cancer therapy, autophagy reportedly promotes either cell survival or death. We showed here that inhibition of autophagy by CQ treatment enhanced apoptosis with caspase-3 activation and impaired Renca cell survival. It seems that autophagy induced by serum deprivation is involved in anti-apoptosis. On the other hand, a high level of autophagy can cause necrotic cell death that is not associated to necroptosis and inactivation of autophagy is related to tumorigenesis [20, 38, 41]. The induction of autophagosome/autolysosome formation was observed only in HCaRG-Renca cells and HCaRG overexpression was associated with a 3-fold increase in the percentage of necrotic cells that was not diminished by the necroptosis inhibitor, Necrostatin-1. Moreover, the presence of multinucleated giant cells was a characteristic of HCaRG-RCCs, and HCaRG overexpression led to the appearance of multinuclear giant cells and over-activated autophagy in these cells (Supplementary Figure 4A and 4B). Altogether, our date suggest that activated autophagy by HCaRG promotes PCD rather than prolong cancer cell survival.

We previously reported that HCaRG levels are decreased in various cancer cell lines including glioblastoma, a partly differentiated RCC and a moderately differentiated hepatocellular tumor [1]. Immnohistochemistry of surgical specimens shows that HCaRG expression is suppressed in ccRCC specimens compared to normal tissues adjacent to tumors. Tumor size is one of the best predictors of RCC prognosis [28], and our study confirm that tumor size and recurrence are higher in patients with low HCaRG levels in normal tissues than in patients with high levels of HCaRG. Numerous studies suggest that biomarkers levels from normal-appearing tissue may be more reliable, due to their homogeneity, compared to the heterogeneous nature of tumors and we are showing here that HCaRG levels in normal tissues correlated with survival rate of patients with ccRCC. In addition, HCaRG secreted form renal epithelial cells inhibited tumor cell proliferation and suppressed the expression of ErbB receptors (EGFR and ErbB3). While the mechanisms of action of secreted HCaRG is still unknown, we have recently observed that intracellular HCaRG controls EGFR internalization and recycling to the cell membrane (Campion *et al.* unpublished data). Schneider *et al.* [42] reported that gene expression levels in tumor-adjacent normal tissue might reflect the regulation of the tumor microenvironment that indicates the possibility of recurrence. Our data suggest that higher HCaRG levels in normal tissue around the tumors could favor controlled cell proliferation and differentiation, thus inhibiting tumor growth and that the screening for HCaRG expression levels and somatic mutations of HCaRG not only in ccRCC but also in normal tissues could be a marker for renal cancerization, progression and prognosis of ccRCC.

Another member of the COMMD protein family, COMMD1, has also been shown to be decreased in a variety of cancers [12]. COMMD1 was shown to inhibit HIF-mediated gene expression, resulting in reduction of tumor metastases. Members of the COMMD protein family are known to interact with one another [43, 44]. We have no evidence that COMMD1 is involved in HCaRG/COMMD5 effects reported here as HCaRG overexpression in Renca cells did not modify COMMD1 expression, but we cannot exclude that HCaRG might interact with other members as it does with COMMD1 in ccRCC (Supplementary Figure 5A and 5B).

In conclusion, this is the first report on the expression and clinical significance of HCaRG in ccRCC suggesting that HCaRG could be marker of development and progression of ccRCC. Our data demonstrate that overexpression of HCaRG inhibits RCC development and tumor angiogenesis by inactivating the proto-oncogene, ErbB2, and gene silencing of EGFR and ErbB3. HCaRG preserves cell differentiation and promotes autophagic cell death. HCaRG/COMMD5 acts as a tumor suppressor gene of RCC and as such restoring its levels to control EGFR/ErbB signaling holds potential to treat cancer.

## MATERIALS AND METHODS

All procedures in this project are conformed to guidelines of the Canadian Council on Animal Care and were approved by the Animal Care Committee of the Research Centre, Centre hospitalier de l’Université de Montréal.

### Studies using human ccRCC samples

The use of ccRCC specimens was approved by the Examination Committee for Clinical Research of Nihon University Itabashi Hospital. The patients underwent surgery for ccRCC at Nihon University Itabashi Hospital (Tokyo, Japan) from 1997 to 2006. The samples were collected from the archives of the Department of Pathology of Nihon University. All patients provided written informed consent prior to surgery. Tumor size was based as the longest diameter of pathological specimens. Kaplan-Meier curves were generated for time to recurrence-free survival in Figure 6C were compared using the log-rank test.

For HCaRG immunohistochemcal staining, sections including not only ccRCC but also normal tissue around tumors were deparaffinized, hydrated, and subjected to antigen unmasking using 10 mM citrate buffer pH 6.0 in a pressure cooker for 5 minutes. After blocking, the sections were incubated with anti-HCaRG-antibody (1:25, 10393-1-AP; Proteintech Group Inc., Chicago, IL, USA) overnight at 4°C. The reaction products were visualized using Cell & Tissue Staining kit (CTS005; R&D systems, Minneapolis, MN, USA) in accordance with the manufacturer’s recommendations. Finally, sections were counterstained with Mayer’s hematoxylin [4].

### Stable transfection and cell culture

Renca , TCMK-1 and B16-F10 cell line were obtained from the American Type Culture Collection (Rockville, MD, USA). Both cancer cells were transfected with control plasmid (pcDNA/Neo1, V79020; Thermo Fisher Scientific, Waltham, MA, USA) or the plasmid encoding rat HCaRG using Attractene transfection reagent (301005; Qiagen, Valencia, CA, USA) following the manufacturer’s protocol. Transfected cells were first selected in G418 (11811023; Thermo Fisher Scientific) containing medium, and single clones were isolated [13]. In some experiments, the cells were serum deprived in Roswell Park Memorial Institute medium (RPMI)-1640 medium or Dulbecco’s modified Eagle’s medium (DMEM) (Thermo Fisher Scientific) in the presence or absence of CQ (036-17972; Wako Pure Chemical Industries, Ltd., Osaka, Japan), Necrostatin-1 (ab141053; abcam, Cambridge, MA, USA), 10 µg/ml of α-amanitin (A2263; Sigma-Aldrich, St. Louis, MO, USA) or 5-Aza-CdR (A3656; Sigma-Aldrich).

### siRNA knockdown

HCaRG was knocked down with siRNA targeting a HCaRG sequence (4390815; Thermo Fisher Scientific). WT-Renca cells were transfected with 25 nM of negative control siRNA (4390844; Thermo Fisher Scientific) or siRNA-HCaRG with HiPerFect transfection reagent (301705; Qiagen), according to the manufacturer’s instructions. Forty-eight hours after transfection, the cells were harvested and knockdown efficacy was assessed by western blot.

### Cell proliferation assay

Stable transfected cells were seeded onto 96-well microliter plates and cell proliferation was measured with the cell proliferation Reagent WST-1 (5015944001; Sigma-Aldrich) according to the manufacturer’s recommendations. The absorption of WST-1 was measured at 450 nm using a Multilabel counter 1420 Victor^3^ V (Perkin Elmer, Wellesley, MA, USA).

### Cell-cycle analysis

Time course of cell-cycle profiles was performed by addition of 10% fetal bovine serum (FBS) medium 24 hours after serum deprivation. Neo- and HCaRG-Renca cells were harvested to proceed to nucleic acid staining. Fixed cells were incubated in phosphate buffered saline (PBS; Thermo Fisher Scientific) containing 0.5 mg/ml bovine pancreatic DNase-free RNase (11119915001; Sigma-Aldrich) and 50 µg/ml PI (P1304MP; Thermo Fisher Scientific) for 20 minutes at room temperature. Cells were analyzed using FACScanto instrument (BD Biosciences, Mississauga, Canada), and compiled with ModFit LT (Verity Software House, Topsham, ME, USA) and FACS DivaSoftware (BD Biosciences).

### Cell death analysis

Plating of Neo- and/or HCaRG-Renca cells on 96-well microliter plates was adjusted so as to reach the same number of living cells at the time of cell death assessment. After serum deprivation, cells were double-stained with calcein-AM and EthD-1 (L3224; Thermo Fisher Scientific) according to the manufacturer’s instructions. Live and dead cells were viewed by a fluorescence microscope (Olympus, Hicksville, NY, USA) and quantified using the microplate reader at wavelengths of 485 nm and 540 nm, respectively.

Apoptosis was detected with the DeadEnd Fluorometric TUNEL system (G3250; Promega). Briefly, after 24 hours of serum deprivation, cells were fixed and permeabilized. After pre-equilibration, strands of DNA were end-labeled by incubation with recombinant terminal deoxynucleotidyl transferase for 60 minutes at 37°C. The reaction was stopped by adding 2× saline-sodium citrate buffer for 15 minutes. After washing, the slides were mounted with Vectashield mounting medium with 4′,6-diamidino-2-phenylindole (DAPI, H-1200; Vector Laboratories, Inc., Burlingame, CA, USA) and viewed by fluorescence microscopy.

Autolysosomes in Renca cells incubated with or without serum for 3 hours were detected by staining with the Cyto-ID Green autophagy detection reagent (ENZ-51031; Enzo Life Sciences Inc., Farmingdale, NY, USA) and LysoTracker Red probe (L7528; Thermo Fisher Scientific) according to the manufacturer’s instructions. After incubation for 30 minutes, cells were fixed in 4% paraformaldehyde and mounted with Vectashield mounting medium with DAPI.

To elucidate dead cell populations using the annexin-V/PI dual-staining assay (556547; BD Biosciences), collected cells were re-suspended in 100 μl of binding buffer containing 5 μl of FITC-Annexin-V and PI for 15 minutes at room temperature in the dark. After incubation, the cells were added to 400 μl of binding buffer and then analyzed using FACScanto instrument.

### *In vivo* tumor growth

BALB/c male mice were purchased from Charles River Laboratories international, Inc. (Montreal, QC, Canada). To generate subcutaneous tumor, 1 × 10^6^ Neo- or HCaRG-Renca cells were injected subcutaneously into the right flank of BALB/c mice under isoflurane anesthesia. Six 8-week old BALB/c mice were used per group. Tumors were measured on twice per week using caliper, and tumor volume (TV) was calculated using the following formula: TV = *A* × (*B*)^2^ × 0.52. *A* and *B* were the longest length and width, respectively, for each tumor. After 4 weeks, the tumors were harvested and stained with H&E.

### mRNA quantification and expression

Real-time quantitative PCR was performed with diluted cDNA using designed Taqman probes (Thermo Fisher Scientific), according to the manufacturer’s instructions. Real-time PCR data were analyzed with standard curves and normalized to glyceraldehyde-3-phosphate dehydrogenase (GAPDH) with its specific primer sets (5′ and 3′ primers: 5′-TGCACCACCAACTGCTTAGC-3′ and 5′-GGCATGGACTGTGGTCATGAG-3′) as described previously [4, 45]. Correlation coefficients for standard curves were all >0.95.

### Western blot

Rat tumor tissues, cultured cells and acetone-precipitated supernatants were lysed in a modified radioimmunoprecipitation assay protein extraction buffer as described previously [4]. Each sample was applied equally to 8–15% polyacrylamide gels and transblotted to polyvinylidene difluoride membranes (GE healthcare, Uppsala, Sweden). After blocking, primary-antibodies to AKT1 (1:2000, 2938), phospho-AKT1 (1:1000, 9271), Caspase-3 (1:1000, 9665), ERK1/2 (1:1000, 4695), phospho-ERK1/2 (1:1000, 4370), LC3B (1:1000, 3868), mTOR (1:1000, 2972), phospho-mTOR (1:1000, 2971), PI3K p110α (1:1000, 249), phospho-PI3K p85/p55 (1:500, 4228) (Cell Signaling Technology, Inc., Danvers, MA, USA), β-actin (1:1000, sc-47778), EGFR (1:250, sc-03), ErbB2 (1:1000, sc-284), ErbB3 (1:1000, sc-285), phospho-ErbB2 (1:500, sc-81508), phospho-ErbB3 (1:250, sc-135654), HIF-1α (1:1000, sc-53546), GAPDH (1:2000, sc-20357) (Santa Cruz Biotechnology, Inc., Santa Cruz, CA, USA), phospho-EGFR (1:1000, 44-790G; Thermo Fisher Scientific), αSMA (1:2000, ab32575; abcam), E-cadherin (1:2000, 610181; BD Biosciences) and HCaRG (1:2000) were incubated overnight, followed by incubation with secondary horseradish-peroxidase conjugated-antibodies (Santa Cruz Biotechnology, Inc.) for 60 minutes. Immunocomplexes were detected by enhanced chemiluminescence (PerkinElmer Life Sciences).

### Immunostaining

Neo- and HCaRG-Renca cells were grown on sterile cover slips under the same conditions described above. For E-cadherin staining, cells were fixed and permeabilized in cold methanol. After blocking, cells were incubated with anti-E-cadherin-antibody (1:100) for 2 hours at room temperature. For αSMA staining, cells were fixed in 4% paraformaldehyde and permeabilized with 0.2% Triton-X-100 in PBS. After blocking, cells were incubated with anti-αSMA-antibody (1:100) overnight at 4°C. For autophagosome assessment, cells were incubated with or without serum for 3 hours. Cells were then fixed, permeabilized and incubated with anti-LC3B-antibody (1:100) for 90 minutes at room temperature. After incubation with secondary-antibodies (Thermo Fisher Scientific), the samples were mounted with Vectashield mounting medium with DAPI and viewed by fluorescence microscopy.

The ccRCC sections were stained using PCNA staining kit (931143; Thermo Fisher Scientific) following the manufacturer’s recommendations, or incubated with anti-Ki-67-antibody (1:25, M7249; Dako, Glostrup, Denmark) or anti-CD34-antibody (1:25, ab8158; abcam). The quantification of CD34-positive microvessels was evaluated in 10 randomly-chosen non-overlapping fields per section for each mouse using Adobe Photoshop CS6 (Adobe Systems Corporation, San Jose, CA, USA) [46]. The results were expressed as the percentage of CD34-positive area in total area of the field.

### Measurement of serum VEGF concentration

Serum VEGF protein levels were determined by a mouse VEGF ELISA kit (KMG0111; Thermo Fisher Scientific) according to the manufacturer’s instructions. Serum samples from mice injected with Neo- or HCaRG-Renca cells under the skin were collected at the end of the study and diluted 1:5 in incubation buffer.

### EGFR, ErbB2 and ErbB3 promoter activities

Human EGFR 5′ flanking region containing 1.7 kb of its promoter, human ErbB2 5′ flanking region containing 0.7 kb of its promoter and human ErbB3 5′ flanking region containing 2.2 kb of its promoter were cloned into multiple cloning sites of pGL4.18 luciferase reporter vector (E6731; Promega). Each reporter plasmid and and pGL4.74-TK vector (E6921; Promega), as an internal control, were used to transfect Renca clones with Attractene transfection reagent. Cells were harvested 48 hours post-transfection and luciferase activities were revealed using a Dual-luciferase reporter assay system (E1910; Promega).

### MassARRAY quantitative methylation analysis

Quantitative analysis of methylation level of EGFR and ErbB3 promoters was performed using a Sequenom MassARRAY Compact System as described previously [47]. Genome DNAs were extracted from cells using Wizard^®^ Genomic DNA Purification Kit (A1120; Promega) according to the manufacturer’s protocol and were treated with sodium bisulfite using the EZ-96 DNA Methylation Gold Kit (D5007; Zymo Research, Irvine, CA, USA). Primers were designed by MethPrimer software to span the promoter CpGi of both genes [48]. Details of primer information are listed in Supplementary Table 1. Bisulfite-treated DNA samples were applied for amplification of the target sequences using HotStarTaq DNA polymerase (203203; Qiagen) with specific primers. PCR products were treated with shrimp alkaline phosphatase and then subjected to *in vitro* transcription and ribonuclease A cleavage for the T-reverse reaction, as described in the manufacturer’s instructions. The samples were incubated with Clean Resin to remove extra salt and spotted on a 384-element SpectroCHIP using a MassARRAY nanodispenser (Samsung, Seoul, Korea), followed by spectral acquisition on a MassARRAY Analyzer Compact MALDITOF MS. The data were analyzed by EpiTYPER software v1.0 (Sequenom, San Diego, CA, USA) to evaluate the quantitative methylation level of each CpG site or an aggregate of multiple CpG sites. The non-applicable reading and its corresponding site were eliminated during calculation.

### Statistical analysis

The animals were randomly divided into Neo- and HCaRG-Renca cells injected groups. Values were reported as mean ± standard error. Values and percentages between groups were analyzed by Student’s *t* test, two-way analysis of variance, χ^2^ test or Fisher’s exact test. All analyses were performed using JMP software v8 (SAS Institute Inc., Cary, NC, USA). *P* < 0.05 was considered to be statistically significant.

## SUPPLEMENTARY MATERIALS FIGURES AND TABLE


