# Supplementary material for: HCaRG/COMMD5 inhibits ErbB receptor-driven renal cell carcinoma
Source: Oncotarget. 2017 May 19;8(41):69559–76. doi: 10.18632/oncotarget.18012 (PMC5642500; doi:10.18632/oncotarget.18012)
Supplement: Supplementary file 1 [file oncotarget-08-69559-s001.pdf]

HCaRG/COMMD5 inhibits ErbB receptor-driven renal cell carcinoma

SUPPLEMENTARY MATERIALS

Supplementary Table 1: Primers for MassARRAY quantitative methylation analysis

Primer sets #	Sequence of primers (5′–3′)
**EGFR #1**	AGGAAGAGAGAGAGAATTATATTGTTGGTGTTGTTGA
	CAGTAATACGACTCACTATAGGGAGAAGGCTCCCTAACAACCCCTCTACTC
**EGFR #2**	AGGAAGAGAGAGTAGGAGTAGAGGGGTTGTTAGG
	CAGTAATACGACTCACTATAGGGAGAAGGCTTCCACACTTCCAAATCATTACTA
**ErbB3 #1**	AGGAAGAGAGAGGTTTAGGGGGAGGGGATTATAA
	CAGTAATACGACTCACTATAGGGAGAAGGCTTTTCCAACCTAAAAACAAACCC
**ErbB3 #2**	AGGAAGAGAGAGGGTTTGTTTTTAGGTTGGAAAT
	CAGTAATACGACTCACTATAGGGAGAAGGCTAATCTCACTCCAACAATTTCC
**ErbB3 #3**	AGGAAGAGAGAGGGAAATTGTTGGAGTGAGATT
	CAGTAATACGACTCACTATAGGGAGAAGGCTACCAAACTAAAAAAAAAACCCA

Tag sequences for EpiTyper analysis are underlined.

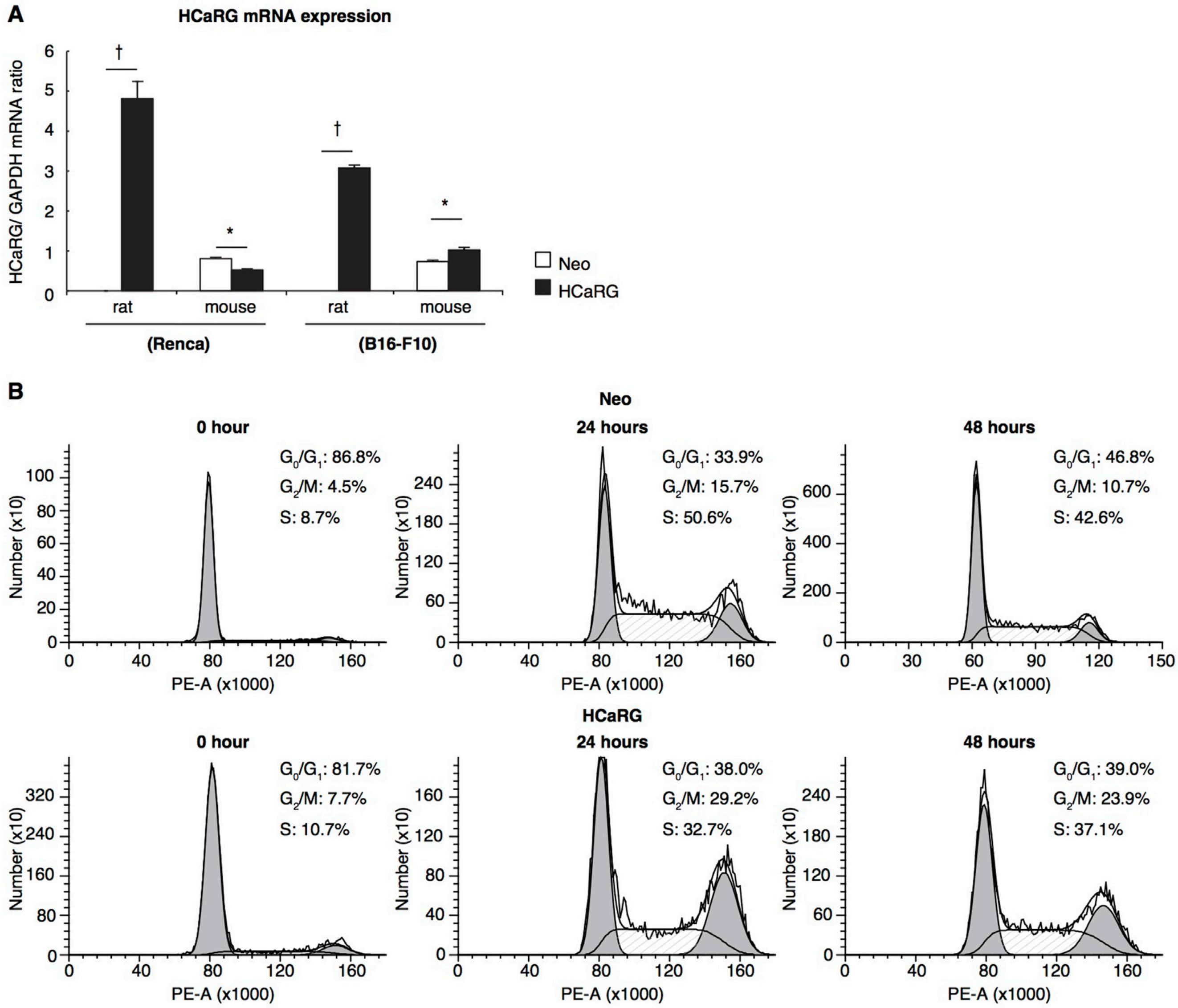

Supplementary Figure 1: HCaRG overexpression delays cell-cycle progression in B16-F10 cells. (**A**) Exogenous rat HCaRG and endogenous mouse HCaRG mRNA levels in Renca and B16-F10 clones were quantified using Real-Time PCR and normalized to GAPDH. Exogenous HCaRG mRNA was detected only in HCaRG-cancer cells. The expression of endogenous HCaRG mRNA was slightly decreased or increased by transgene. (**B**) Representative cell-cycle DNA histograms showed the effect of HCaRG overexpression on cell-cycle progression in B16-F10 cells. HCaRG clearly delayed cell-cycle progression in association with G_2/M accumulation. The cell-cycle length lasted for about 48 hours in Neo-B16-F10 cells and for at least 48 hours in HCaRG-B16-F10 cells. G_0/G_1, G_2/M and S phases are given.

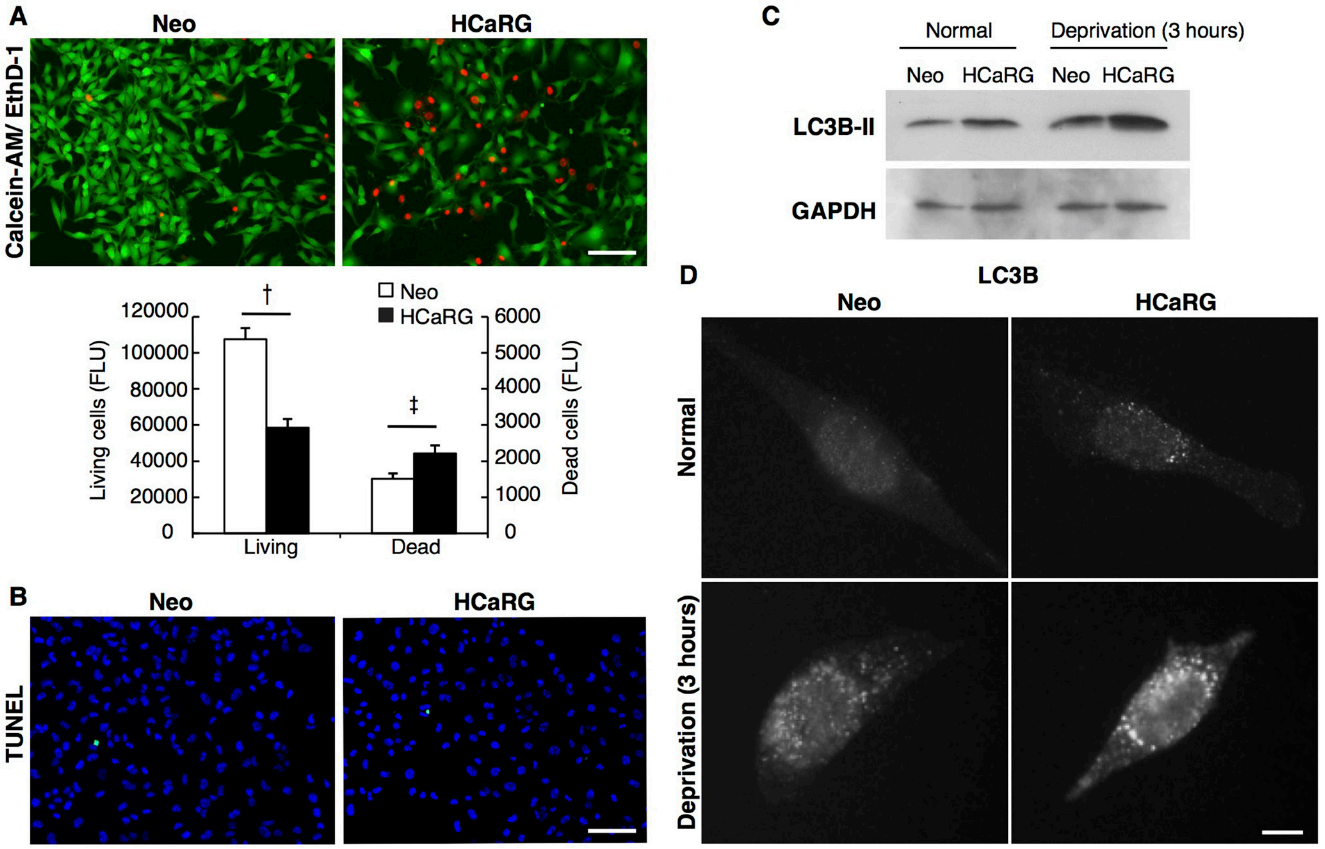

Supplementary Figure 2: HCaRG induces cell death with autophagy distinct from apoptosis in B16-F10 cells. (**A**) Living and dead cells were double-stained with calcein-AM (green) and ethidium homodimer (EthD)-1 (red) for relative fluorescence (FLU) quantification. HCaRG overexpression markedly decreased the number of living cells and increased the number of dead cells compared to Neo-controls 48 hours after serum starvation. ‡*P* < 0.05, †*P* < 0.005. Scale bars, 100 μm. (**B**) TUNEL staining in B16-F10 clones under serum deprivation for 48 hours. Only a few apoptotic cells could be detected without any difference between Neo- and HCaRG-B16-F10 cells. Scale bars, 100 μm. The expression of LC3B, a marker of autophagosome, was demonstrated by western blot in (**C**) and immnunostaining in (**D**) with or without serum deprivation. HCaRG overexpression markedly fostered autophagy under the both conditions as is the case of Renca cells. Scale bars, 10 μm.

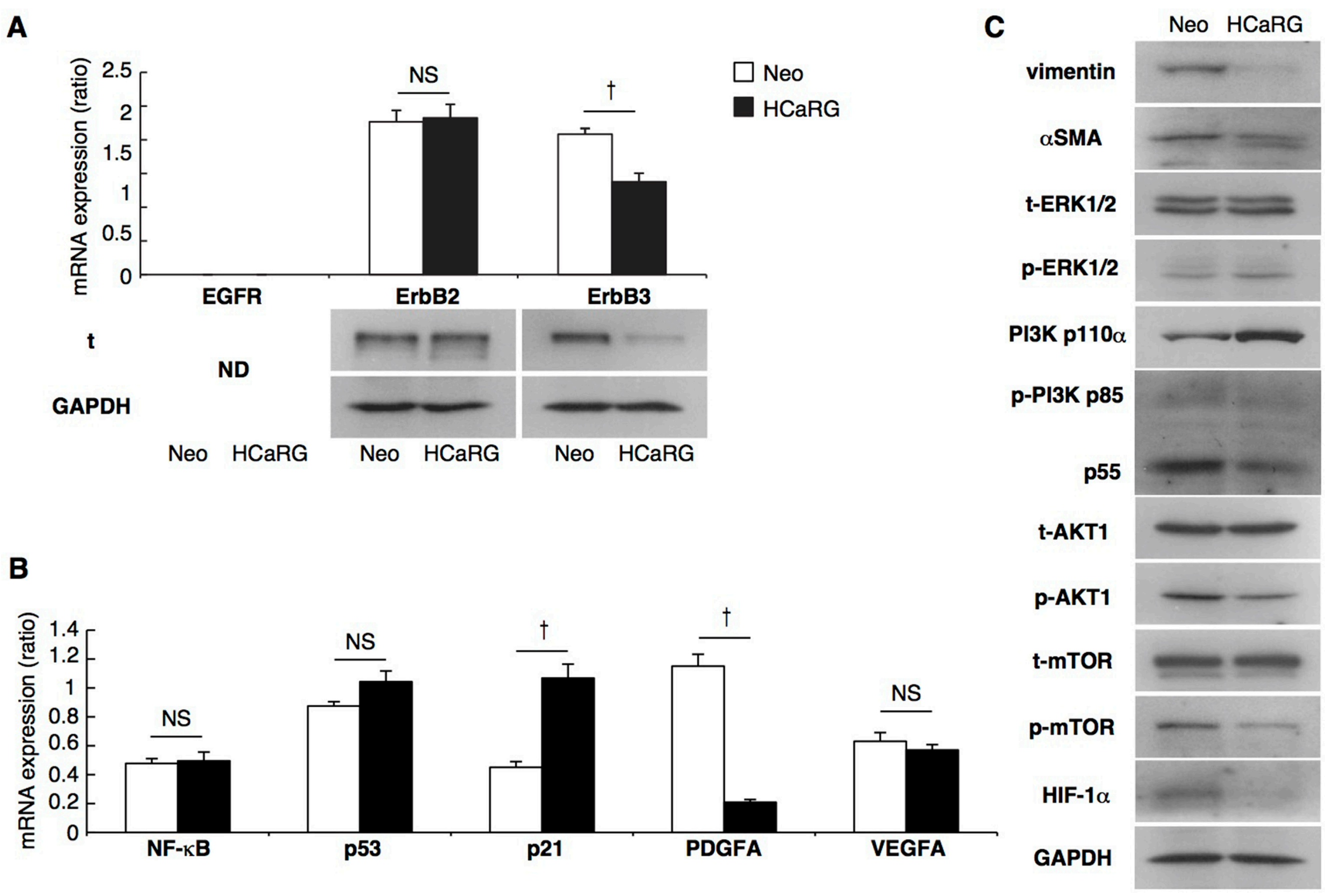

Supplementary Figure 3: HCaRG inhibits the MAPK and PI3K/AKT signaling pathways in B16-F10 cells. (**A**) The expression of total (t) ErbB receptors was demonstrated by Real-Time PCR and western blot in B16-F10 cells. HCaRG overexpression reduced ErbB3 expression at the mRNA and protein levels in HCaRG-B16-F10 cells. ErbB2 mRNA and total protein levels were not modified by HCaRG overexpression. EGFR mRNA and protein were not detectable in this cell line. $^{\dagger}P < 0.005$. ND, not detectable. NS, not significant. (**B**) The effect of inhibition of MAPK and PI3K/AKT signals on downstream genes was demonstrated by Real-Time PCR in B16-F10 cells. $^{\dagger}P < 0.005$. NS, not significant. (**C**) B16-F10 cell lysates were analyzed by western blot using appropriated antibodies. Representative blots show the inactivation of subsequent MAPK and/or PI3K/AKT signaling pathways by HCaRG overexpression.

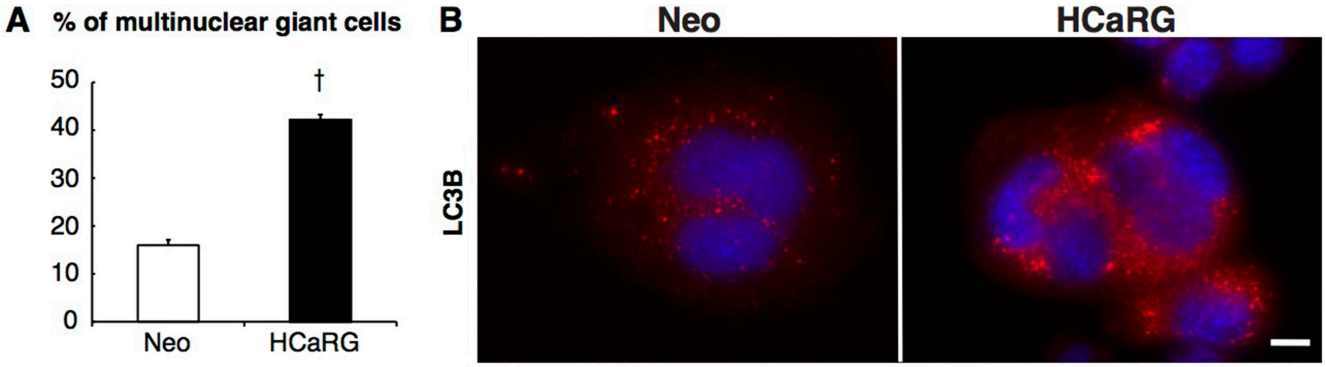

Supplementary Figure 4: HCaRG increases overactivation of autophagy in multinucleated giant cells. (**A**) HCaRG overexpression increased the percentage of multinuclear giant cells as shown by cellular DNA content detected by flow cytometry. $^{\dagger}P < 0.005$. (**B**) Immunostaining for LC3B in Renca clones. The LC3B puncta (red) were increased in multinucleated giant cells of both Neo- and HCaRG-Renca clones compared to respective mononucleated cells. Autophagy was particularly overactivated in multinucleated HCaRG-Renca cells compared to its Neo-control cells. DAPI staining was used to determine nuclei (blue). Scale bars, 10 µm.

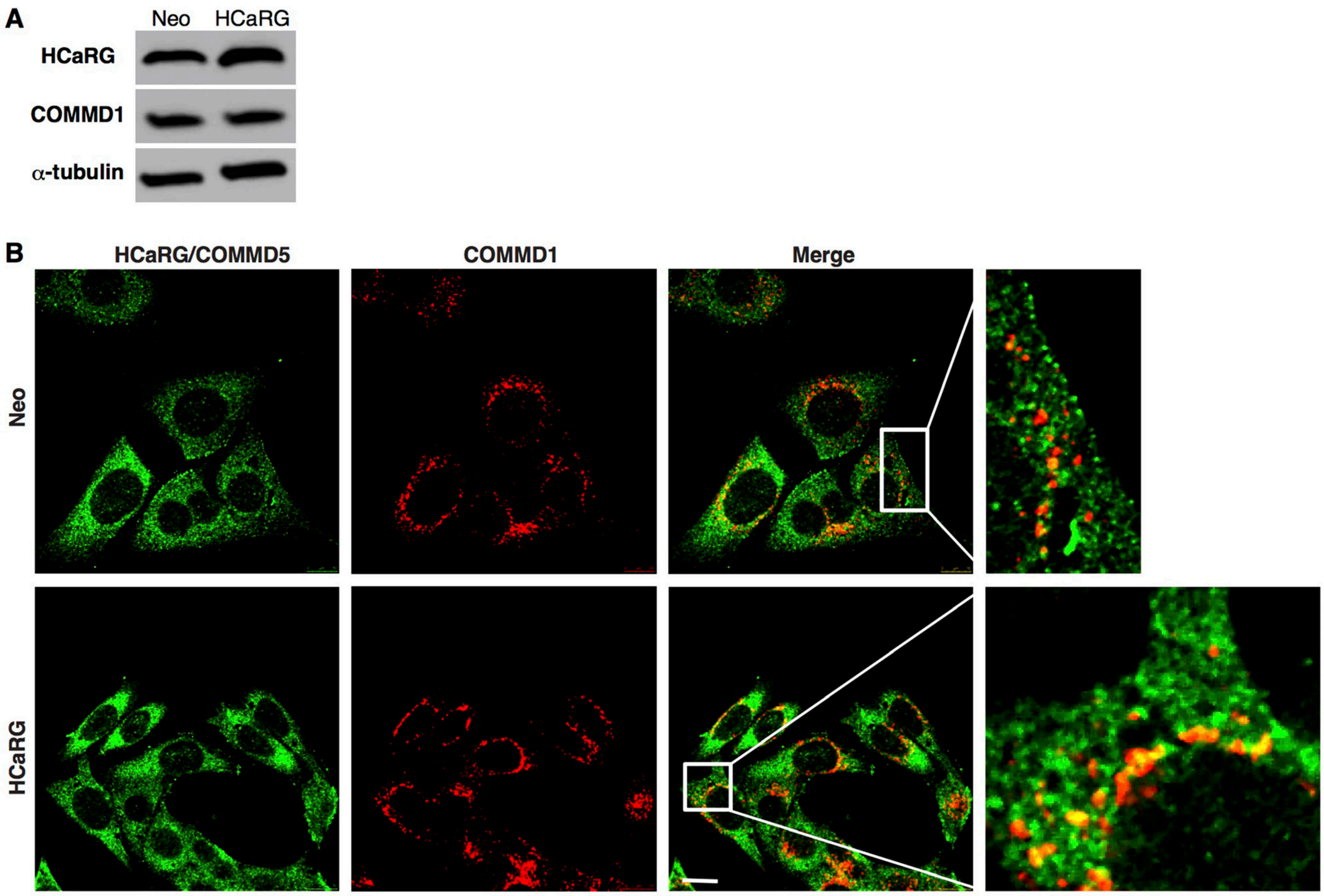

Supplementary Figure 5: HCaRG and COMMD1 expression in Renca cell lines. (**A**) HCaRG overexpression did not modify COMMD1 expression in Renca cells. (**B**) Immunofluorescence of HCaRG (green) and COMMD1 (red). HCaRG was abundant in cytoplasm and plasma membrane and COMMD1 was restricted to the perinuclear region where it colocalized with HCaRG. Neo- and HCaRG-Renca cells were grown to semi-confluence on sterile cover slips. Cells were fixed in 4% paraformaldehyde and permeabilized with 0.1% Triton-X-100 in PBS. After blocking, cells were incubated with primary-antibodies (anti-COMMD5, 1:50 YF-PA26072: AbFrontier, Seoul, Korea and anti-COMMD1, 1:50, 11938-1-AP; Proteintech Group Inc.). After incubation with secondary-antibodies, the samples were mounted and viewed by a Leica laser scanning microscope (Leica Microsystems, Concord, ON, Canada). Scale bar, 10 µm.
